# The Consumptive Curers of New York

**Published:** 1864-04

**Authors:** 


### THE CONSUMPTIVE CURERS OF NEW YORK.

By an Invalid M. D.

With regard to life it has been said by a late writer, that "we persuade ourselves that it teems with novelties and delight; that it abounds with high festival days and gala shows, somewhere in happier regions, although they come not to us." This remark is especially true with regard to the hopes and expectations of the invalid.

Art may fail him at home, measures which he must feel are well directed, may disappoint, friends may mournfully walk around him; still his mind at times overleaps all, and loves to revel in the idea that somewhere, in some unknown land, there lives the mind to conceive, and there exists the remedies which it can direct, for his recovery.

The sweet solace of the mind, Hope, as every one knows, is the constant attendant on consumption, where it is, indeed, a heavenly visitant. Wasting day by day, who has not seen the wretched victim letting go the greater hopes of yesterday, which may have pointed him to a complete recovery, but to cling the faster and with as sweet content to those of to-day, although they promise only a partial convalescence.

I cannot conceive of a more beautiful dispensation of Providence than this. Round and round in a narrowing circle, day by day, but nearer the end, yet there is always hope that the last thing tried, despite of preceding failures, will prove just what is wanting. So sweet a comfort the pitying angel must send for a good purpose. But so much good comes not unmixed with evil; for this very buoyant feeling of hope is taken advantage of by designing men, whose promises to furnish remedies to suit every case, are only equalled by the extent of the popular credulity. I believe that it is in ignorance, that such deceive the afflicted. If there are any who do it knowingly, who *will* take advantage of this heaven-born feeling for the purpose of money-making; to them I have but to say with Othello:

> Never pray more: abandon all remorse,
> *  *  *  *  *  *  *
> For nothing canst thou to damnation add,
> Greater than that.

Messrs. Editors, guided by just such feelings as I have attempted to describe, I directed my footsteps to the great city of New York. I have a large cavity in the upper part of the left lung, and I had been told with a sad voice and a firm aspect, by one whom I loved and had every reason to respect, that softening had already begun in the apex of the opposite organ; and I am emaciated to the last degree. Nevertheless, from the glowing accounts which I had heard of the wonderful power over the disease, possessed by numberless men in this great commercial emporium of our Union, I resolved to proceed thither at all hazards.

My mind was filled with vague, but most embarrassing hopes, shadowy outlines of superhuman skill, in men, high above their fellows in point of pure humanity and disinterested devotion to the science of life, flitted before my morbid vision, giving me strength to endure the journey.

Two great parties I found engaged with equal zeal in this important work. The one I shall describe as the constitutional class, or those who adopt a general treatment; the other the local, or those who adopt a strictly topical method of cure. I had been educated in the former school, and did not tarry long with its professors.

Improve the nutrition,—cod liver oil—good diet—much out door exercise,—measures which I knew had saved me so far, were all they could tell me about, but knowing all this before, I was not satisfied, and wished to go farther; for these I found were not the men who were doing so many wonders. I diverged a little into the intermediate class—a sort of divining doctor, by spiritual agency—who had an immense run, as I learned, among the clergy. The Dr. was overrun with patients—his ante-room was like the lobby of a theatre on the night of a popular actor's benefit.

I took my seat, and abiding my turn, it came at last. I found behind the scenes, one having the air of "a most prosperous gentleman," who looked through my case with an imposing flourish—smiled approvingly—received a fee,—I thought an enormous one,—and bade me follow him and I would be well. Conducting me back into the ante-room, he sang out some words in an unknown tongue to a clerk near the window, who wore a remarkably stiff, standing shirt collar, and then, with a graceful wave of the hand, withdrew. This latter person at once handed me a package of medicine, already neatly put up in a handsome paper box. Ah, said I in surprise, did you have it ready? Yes, said the clerk, carelessly, I saw you come in, and whilst you were waiting to see the Boss, I put it up. Then, rejoined I with warmth, you knew beforehand what he intended to give me?

The clerk with a stiff standing collar, thrust his tongue into his left cheek, drew the lower lid of his right eye grimly down with his ring finger, leered at me significantly, but with much good nature, and I departed, I trust a wiser man.

After visiting a man who had told me that he had enjoyed the honor (hitherto unknown to Americans) of being the physician for many years to her majesty, Victoria, queen of England, and that he had a book which he sold for 12½ cents, which would tell me how to cure myself of any disease as well as he could; and which I did not buy for reasons which must be obvious; I became disgusted with this whole class, and having no other alternative, threw myself into the arms of a Topical party, with hope still undiminished.

But in this there was some difficulty, for I found two parties, and which to select perplexed me some. The one I shall characterize as the *Probangers*, the others the *Inhalers*. From what I learned, the history of these parties is possessed of no little interest.

It appears that the Probang, and its accompanying sponge and caustic, were not originally used to cure consumption. In simple throat affections, however, it had had an immense run. Clergymen every where had tried it, and such of their flocks as they could influence had followed suit, and the whole thing became rapidly much in vogue.

Finding how easily it went down the throats of the people, it bye and bye took a bolder stand, and stoutly proclaimed its power to cure consumption, in its most common form. Still, it must be remembered, in all this time it never claimed to go beyond the bifurcation. But we all remember how popular it was, and what vast sums of money it must have made.

Whether it was the latter, which is a great stimulus to invention, or some higher motive, it is certain that this thing did not pass unnoticed. Active minds were at work, and vigorous intellects became engaged in tapping this rich mine, and in pushing farther the investigation. As the result of all this, inhalation turned up. The probangers were taken on their own ground—the people were told that if topical treatment, so partially applied, was successful, how much more were they entitled to expect from a method making the whole lung accessible to medical agents. The reasoning was plausible—the thing took—Probangdom tottered to its very base, and inhalation became the rage.

But our friends were not to be put down in this manner; they were penetrating men, and at once saw that all they had to do was to go a little deeper. The old idea of the bifurcation being the limit of the probang was therefore abandoned, and it was proclaimed that cavities could be invaded and sponged out, and that inhalation could not dare do more than it.

This was the state of things at the time of my visit, and it was this which led to my perplexity already spoken of.

But as I had already (as every body else almost have done in my situation) used the probang, as far as the bifurcation, as it was said to have been applied, I at length decided upon inhalation, and repaired without delay to its head-quarters in the city. The Doctor received and examined my case with exceeding care. At home, my medical friends could perceive at a glance, as they told me, by the flattening of the left side of the chest, and by its quiescence during respiration, the nature of my disease, but these signs were not sufficient for my new adviser.

He stripped me to the skin, measured, percussed, and auscultated, over and over again every part. I never saw so much pains, and would have thought some of his manipulations indicative of decided "greenness," but for the exalted reputation and obviously large experience of the operator. He found my case a very beautiful one—I was, he said, just enough diseased to test the full power of his method. In the course of his remarks, however, it turned up that this person was not the genuine man so widely known, and I dressed myself with some show of indignation. He took my complaints very quietly, and showed me into the next room. The person who there received me won my heart. He showed me around, examined my case, predicted "a good time coming" for me soon, but in the midst of it all, announced himself as only an assistant, and appeared greatly surprised that I should think anything of that. I stamped in rage, and announced that I

had come all the way from Virginia just to see the genuine article, and would not be satisfied with any substitute. This gained me admission into the great man's presence. I found him superb. My account of my reception amused him much, and we became unreserved and quite intimate.

The fact was, he told me, that these fellows had come well recommended to him,—the business had prospered in their hands—he was no judge of qualification—didn't pretend to it—had seen an opening for it, had got the business up, and managed only the advertising and money department. In short, said he, I am only the capitalist of the concern. The little fellow you first saw, he furthermore proceeded, is I think myself, a little too fussy over the chest, but the other one, I do think, is *nice* for the throat.

I had one other chance, which was to have my cavity sponged out. The Doctor told me my case was a beautiful one for the treatment. I admired his ingenious arguments in favor of his method, and was quite carried away by his charming description of the *rationale* of the whole operation. When he finished, I announced, with enthusiasm, that I was a convert to his views, and pronounced myself ready at any moment for the operation. He examined me again with greater care, and with a faint touch more of gravity in his countenance. It was just the thing for my case, said he, and would have to be done, but not then. You get back home, he proceeded, and *get a little more strength*, and then return, and I'll perform the operation for you.

One hour afterwards, feeling as a doomed man, I left the great city of New York. The consumption curers have taken from me all my bright hopes, and left me but a mockery.—*Virg. Med. and Surg. Jour.*

